# Exploring the Role of Place in Rural Older Adults’ Social Isolation: A Life-Course Perspective

**DOI:** 10.1093/geroni/igaf122.1714

**Published:** 2025-12-31

**Authors:** Pallabi Bhowmick, Erik Stolterman Bergqvist

**Affiliations:** University of Illinois Urbana-Champaign, Champaign, Illinois, United States; Indiana University Bloomington, Bloomington, Indiana, United States

## Abstract

Loneliness among older adults is a public health concern, significantly affecting their mental health and well-being. While technology interventions to foster social connections have been widely studied, the role of place – a specific location or community – remains underexplored. This interview study conducted with 12 older adults, aged 66-85 (M: 75.2; SD: 7.1) applies a life-course perspective to explore how older adults’ perceptions of place, shaped by personal histories, social networks, and environments, contribute to feelings of loneliness. Focusing on a rural community in Indiana, USA, the study examines how both physical and social aspects of place interact with aging to either foster or alleviate loneliness. Preliminary findings suggest that rural settings often contribute to loneliness due to geographic distance from neighbors/family and limited access to services. However, the sense of belonging and community found in these rural settings, rooted in long-standing social networks and practices, can reduce loneliness. The study further reveals that older adults in rural areas, particularly those at different life-course stages, may experience loneliness in varying ways. For instance, older individuals who have lived in the community for decades may have stronger social ties, while newcomers or those with fewer local connections may experience more loneliness. By applying a life-course perspective, this research underscores the importance of understanding how aging and place intersect over time. The findings offer insights into how technology can bridge social gaps created by geographic isolation, fostering social engagement and enhancing the quality of life for older adults in rural communities.

